# Intracranial foreign body (bullet) during pregnancy

**DOI:** 10.4274/tjod.08860

**Published:** 2014-12-15

**Authors:** Ali Akdemir, İsmet Hortu, Burak Zeybek, Asuman Sargın, Niyazi Aşkar

**Affiliations:** 1 Ege University Faculty of Medicine, Department of Obstetrics and Gynecology, İzmir, Turkey; 2 Ege University Faculty of Medicine, Department of Anesthesiology and Reanimation, İzmir, Turkey

**Keywords:** Intracranial foreign body (bullet), high-risk pregnancy, cesarean section

## Abstract

Intracranial foreign bodies during pregnancy is a very rare condition, however its maternal and fetal outcomes are very crucial with regard to morbidity and mortality. Furthermore wounding by firearms is still a public health problem particularly in our country. Intracranial foreign bodies during pregnancy is high risk pregnancy and must be managed with care and multidisciplinary approach. During this course and labour avoiding the increase in intracranial pressure is the most important key point. In this case report we present the follow-up and outcome of a patient with bullet in brain after intracranial injury caused by firearm.

## INTRODUCTION

Intracranial space-occupying formations are very rare in pregnancy^(1)^. In the process of adaptation to pregnancy, physiological changes in the body occur in many systems. These changes may cause the growth of a neoplastic formation in the intracranial area or the increase of intracranial pressure. In the same way, foreign bodies detected in the intracranial area (shell and shrapnel fragments related to injuries due to firearms etc.) may also cause maternal and fetal morbidity and mortality by increasing the intracranial pressure during pregnancy as well as during childbirth. For this reason, it is necessary to avoid interference and manipulations that may increase the patient’s intracranial pressure during pregnancy and childbirth.

## CASE

**Figure 1:**
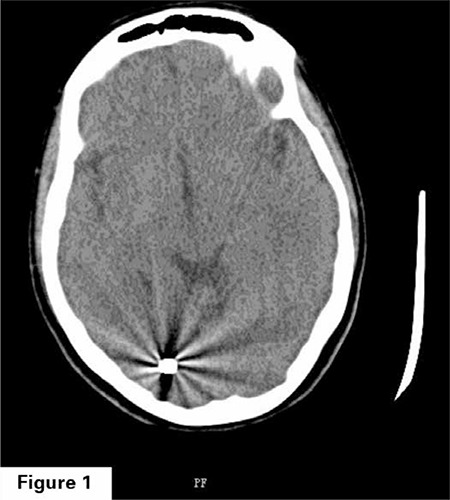
A bullet with 9x6 mm can be seen in the left posterior parieto-occipital area in the cranial CT horizontal cross-section

**Figure 2:**
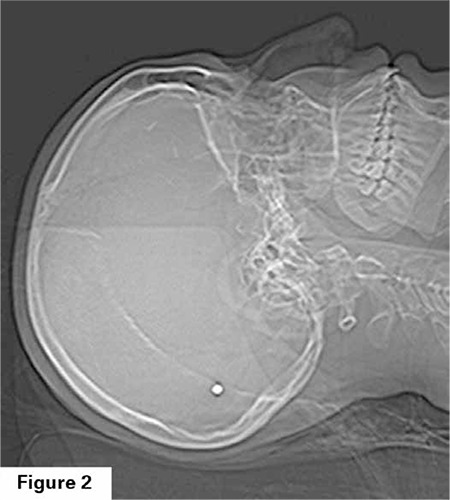
A bullet with 9x6 mm can be seen in the left posterior parieto-occipital area in the cranial CT sagittal cross-section

A twenty-seven year old gravida 2 parity 1 patient applied to our clinic to get pregnancy follow up at the 8^th^ week of her pregnancy. In her past history, it was stated that she had a craniotomy operation since she had a bullet in the cranium due to a firearm injury from a close distance eight years ago. During the patient’s physical examination, it is found out that the injury was in the left frontal bone and there is a secondary defect to the operation, and the motion of the right eye is minimally limited. Despite the serious injury of the patient, luckily there was not a significant neurological deficit. She expressed that within the postoperative first 3 months, she had sight impairment, but afterwards the problem with her vision has improved. When her previous cranial imaging methods examined, by Computed Tomography (CT), it is observed that in the cranium posterior at the left parietooccipital area there was a bullet-compatible mass with 9x6 mm in diameter with high signal intensity within brain tissue (Figure 1, 2). Followed by neurosurgery clinic during pre-pregnancy, it is learned that the patient have had no neurological complications or sequels during follow-up.

By obstetric ultrasound examination, an embryo with 8-9 weeks with the localized heart rate of intrauterine was found. Subsequent follow-up of the patient has been conducted in our clinic during the pregnancy. There has been no obstetric or other systemic problems encountered. The patient has been consulted at regular intervals throughout pregnancy and at the 39^th^ week she was examined by neurosurgery physicians, and their suggestions were taken into consideration. In line with the recommendations of the neurosurgery consultant physicians, elective cesarean delivery was determined in order to avoid increased intracranial pressure and the risk of a possible cerebral herniation, intracranial hemorrhage, and convulsions as much as possible. The patient has also been consulted by the anesthesiology physicians and due to the risk of intracranial pressure changes and herniation, it is determined to perform the operation under general anesthesia instead of regional anesthesia (spinal, epidural). After receiving written informed consent from the patient, to avoid the risk of aspiration, ranitidine and metoclopramide intravenous (iv) were administered 30 minutes before anesthesia. At the time of operation, heart rate (HR), noninvasive blood pressure and peripheral oxygen saturation (SpO_2_) have been monitored and on the back of the left hand vascular access was established by a 18-gauge (G) cannula and through iv, isotonic saline solution was given. After preoxygenation at induction with 100% oxygen, 10 μkg-1 atropine, 4 mg kg-1 thiopental, 0.6 mg kg-1 rocuronium and 0.5 μ kg-1 remifentanil were administered and by applying 1% sevoflurane in 50% air/O_2_ endotracheal intubation, the patient was supported by the mechanical ventilator. Live baby girl of 2510 g was delivered with cesarean section. Baby’s 1^st^ and 5^th^ minute Apgar scores were 8 and 10 respectively. The patient’s arterial blood pressure remained stable during follow-up and end tidal CO_2_ (EtCO_2_) was maintained at 30-35 mmHg. After the end of the operation the patient was extubated at 0.5% sevoflurane. The patient was transferred to the intensive care unit as in the state of conscious, cooperative, and stable vital signs. No problems were monitored in the postoperative period in our 24-hour monitored patient. The patient was discharged home on the 2^nd^ postoperative day with recommendations.

## DISCUSSION

Firearm injuries, as well as all over the world, especially in our country in terms of mortality and morbidity is a major public health problem^(2,3)^. Despite all the legal sanctions, socio-culturally the habit of possession and use of firearms results in death or damages causing injuries both intentionally or accidentally.

Neoplasms or foreign bodies in the cranium during pregnancy are extremely rare. According to the literature, although it is seen that there are many articles about fetal injuries due to firearms during pregnancy, the foreign bodies related to maternal intracranial gunshot wounds or the sharp object traumas haven’t been reported. In this regard, we think that the case above will contribute to the literature. The literature has reported cases of gunshot wounds in pregnancy and the phenomenon that the trauma and foreign bodies cause injuries more on maternal extremity, abdominal organs, uterus^(4,5)^. The study of Wilson et al.^(6)^ at which they examined six pregnant women whose two fetus can be kept alive were injured by firearms during wartime, and the study of Lin et al.^(7)^ at which they examined 13 pregnant women, among whose fetuses only two survived and other pregnancies resulting in death, were wounded by gunshot are in the literature as a series of studies. The researchers expressed the importance of the issue by presenting their case reports in these rare situations. Our case is important in terms of follow up and delivery management as the patient has been exposed to cranial gunshot injury before the pregnancy and the bullet being present the patient got pregnant so it is classified as a high-risk pregnancy.

The researches have shown that, bullet left in the body or pellet residues after gunshot injuries increase blood lead levels and as a result besides the long-term effects, in the short-term they may cause chronic abdominal pain, vomiting, and anorexia^(8,9)^. In our case, any complications or complaints were not noted related to lead toxicity or intracranial pressure changes during pregnancy and as well as before pregnancy. When there is an intracranial foreign body at pregnancy, another aspect to be considered is that a possible intracranial pressure increase which is secondary to pain and straining during the delivery may cause foreign body’s parenchymal damage, intra-cerebral hemorrhage, cerebral herniation and its related sequel. Another thing to be avoided in such cases is Magnetic Resonance Imaging (MRI) that has intense magnetic fields of imaging method. Because the intracranial localized metal objects can be mobilized in the intense magnetic environment and can lead to complications up to some of morbidities and even mortality.

Type of delivery in pregnant women with intracranial foreign bodies is another important issue to be focused on. In order to reduce the intracranial pressure to a minimum levels, these patients should have caesarean section in terms of maternal and fetal well-being. In cesarean section anesthesia, general and regional anesthesia (spinal, epidural, combined spinal epidural anesthesia) techniques are used. Although most of our patients prefer regional anesthesia, in cases such as our patient where it is contraindicated, it is required to have general anesthesia^(10,11)^. Pulmonary aspiration, difficult airway and the rise of blood pressure and intracranial pressure during intubation are particularly among the most important risks of general anesthesia. Therefore about 15 to 20 minutes before the surgery, administration of 30 ml of 0.3 M sodium citrate or alternatively H_2_ receptor antagonist may help by raising gastric pH and may reduce the risk of pulmonary aspiration. In addition, due to the risk of pulmonary aspiration during anesthesia in pregnant women, the induction needs to be done quickly. In our case, aspiration prophylaxis was administered by giving both H_2_ receptor antagonist and metoclopramide before the operation. For rapid induction, 1 mg kg-1 of rocuronium was administered. During intubation, opioid administration is preferred because of the possibility of hemodynamic instability. Using balanced anesthesia with sufficient depth, it is aimed to maintain hemodynamic stability by minimizing the possible sympathetic activation. In our case, a stable hemodynamic status was achieved with the general anesthesia. PaCO_2_ 25-30 mmHg hyperventilation reduces the intracranial pressure, but it causes deterioration on the utero-placental perfusion and may harm fetus^(12)^. In our case, therefore, during follow-up end-tidal CO_2_ levels was maintained at 30-35 mmHg.

As a result, it can be said that presence of intracranial foreign bodies during pregnancy is a rare situation, but in our country gunshot injuries still remains a major public health problem. The case we presented, follow-up and management of other similar patients are extremely important in terms of reducing maternal and fetal morbidity.
